# Deep Learning Models for Histopathological Classification of Gastric and Colonic Epithelial Tumours

**DOI:** 10.1038/s41598-020-58467-9

**Published:** 2020-01-30

**Authors:** Osamu Iizuka, Fahdi Kanavati, Kei Kato, Michael Rambeau, Koji Arihiro, Masayuki Tsuneki

**Affiliations:** 1 Medmain Inc., Fukuoka, 810-0042 Japan; 2 Medmain Research, Medmain Inc., Fukuoka, 810-0042 Japan; 3 School of Medicine, Hiroshima Uniersity, Hiroshima, 734-0037 Japan; 4 Department of Anatomical Pathology, Hiroshima University Hospital, Hiroshima, 734-0037 Japan

**Keywords:** Gastric cancer, Classification and taxonomy, Colon cancer, Gastric cancer, Colon cancer

## Abstract

Histopathological classification of gastric and colonic epithelial tumours is one of the routine pathological diagnosis tasks for pathologists. Computational pathology techniques based on Artificial intelligence (AI) would be of high benefit in easing the ever increasing workloads on pathologists, especially in regions that have shortages in access to pathological diagnosis services. In this study, we trained convolutional neural networks (CNNs) and recurrent neural networks (RNNs) on biopsy histopathology whole-slide images (WSIs) of stomach and colon. The models were trained to classify WSI into adenocarcinoma, adenoma, and non-neoplastic. We evaluated our models on three independent test sets each, achieving area under the curves (AUCs) up to 0.97 and 0.99 for gastric adenocarcinoma and adenoma, respectively, and 0.96 and 0.99 for colonic adenocarcinoma and adenoma respectively. The results demonstrate the generalisation ability of our models and the high promising potential of deployment in a practical histopathological diagnostic workflow system.

## Introduction

According to global cancer statistics^1^, stomach and colon cancers are amongst the most common leading causes of cancer deaths in the world, with stomach cancer ranking fourth in men and seventh in women, and colon cancer ranking third in men and second in women. In 2018, there were 1,033,701 new cases and 782,685 deaths due to stomach cancer, and 1,096,601 new cases and 551,269 deaths due to colon cancer.

In routine clinical pathological diagnosis, histopathological examination of specimens (e.g.haematoxylin and eosin (H&E) stained glass slides) is conventionally done under light microscopy. Whole slide images (WSIs) are the digitised counterparts of glass slides obtained via specialised scanning devices, and they are considered to be comparable to microscopy for primary diagnosis^2^. The advent of WSIs led to the application of medical image analysis techniques, machine learning, and, more recently, deep learning techniques for aiding pathologists in inspecting WSIs and diagnosing cancer. In particular, deep convolutional neural networks (CNNs) have shown state-of-the-art results in a large number of computer vision^3,4^ and medical image analysis applications^5^. Promising and successful computational pathology applications include tumour classification and segmentation, mutation classification, and outcome prediction^6–17^. These results highlight the potential large benefits that could be obtained when deploying deep learning-based tools and workflow systems to aid surgical pathologists and support histopathological diagnosis, especially for increasing primary screening efficiency and diagnostic double-reading.

One of the main challenges in computational pathology is the sheer size of a WSI. A single image obtained at 20X magnification can contain several billion pixels, while the area of interest can be as small as a few thousand pixels. To apply a deep learning classifier, the WSI has to be divided into several thousand tiles, with the classifier then applied independently on each tile. The output from all of the tiles then needs to be aggregated to obtain a final WSI classification. Another challenge that results from this is the time-consuming task of obtaining a large dataset of WSIs with detailed pixel-wise annotations, making it difficult to train a supervised deep learning classifier with a large variety of images. An alternative to supervised learning is weakly-supervised learning, where, instead of requiring pixel-wise annotations, only the whole-slide diagnosis labels are used. Such a technique would require a significantly larger number of WSIs and computational resources. This has been attempted recently by Campanella *et al*.^17^ with impressive results obtained on a dataset of 44,732 WSIs. Deep learning has been previously applied to WSIs of stomach and colon, albeit to small datasets (11–500). For stomach cancer, Sharma *et al*.^18^ used a dataset of 11 WSIs to perform carcinoma classification. For colon cancer, deep learning has been used for predicting survival outcomes^19,20^, classification of nuclei^21^ and polyps^10,22^.

Here, we propose deep learning models to classify epithelial tumours (adenocarcinoma and adenoma) of stomach and colon for supporting routine histopathological diagnoses by surgical pathologists. In the present study, we have collected two large datasets of stomach and colon consisting of 4,128 and 4,036 WSIs, respectively. The collection of WSI were obtained from Hiroshima University Hospital (Hiroshima, Japan) and Haradoi Hospital (Fukuoka, Japan) and manually annotated by pathologists. We then trained a CNN based on the inception-v3 architecture^23^ for each organ, using millions of tiles extracted from the WSIs, to classify a tile into one of three labels: adenocarcinoma, adenoma, and non-neoplastic. We then aggregated the predictions from all the tiles in a given WSI to obtain a final classification using two strategies: a simple max-pooling approach and a recurrent neural network (RNN).

## Results

### A deep convolutional network for discriminating WSI histopathology

The aim of this study was to develop deep learning models that can detect and discriminate epithelial tumours (adenocarcinoma and adenoma) in biopsy specimen WSIs of stomach and colon. The bulk of our dataset originated from Hiroshima University Hospital (Hiroshima, Japan), with 4,128 stomach WSIs and 4,036 colon WSIs. The rest of our dataset, consisting of 500 stomach WSIs and 500 colon WSIs, originated from Haradoi Hospital (Fukuoka, Japan). The Hiroshima University Hospital cohorts were randomly split, in a stratified fashion, on a WSI level, to obtain 500 WSIs for the test set of each organ, with the remaining used for training and validation (5%). The Haradoi Hospital cohorts were not used for training and were reserved exclusively to be used as independent test sets. While the stomach training set consisted solely of biopsy images, the colon training set contained a few surgical resection cases (5%). In addition, we obtained independent stomach and colon surgical resection cases from the publicly available repository of The Cancer Genome Atlas (TCGA) program^24^ to use as test sets.

We used the inception-v3 network as the architecture for our models, and trained them from scratch. We used it as it has been previously shown to work well on WSI classification tasks^12,25^. The models were trained on 512 × 512 pixel tiles that were randomly extracted from the training sets at a magnification of 20X. Each tile was assigned one of three labels: adenocarcinoma, adenoma, or non-neoplastic. Further data augmentations in the form of tile rotations and colour shifts were performed during training to increase robustness and add regularisation to the network. During inference, the trained networks were used in a sliding window fashion with input tiles of 512 × 512 pixels and a fixed stride that is smaller than the input tile size. A sliding window was used because all tiles need to classified to obtain a WSI classification. Smaller strides result in finer heatmaps and longer inference times, while larger strides result in coarser heatmaps and shorter inference times. We used a stride of 256.

**Figure 1:**
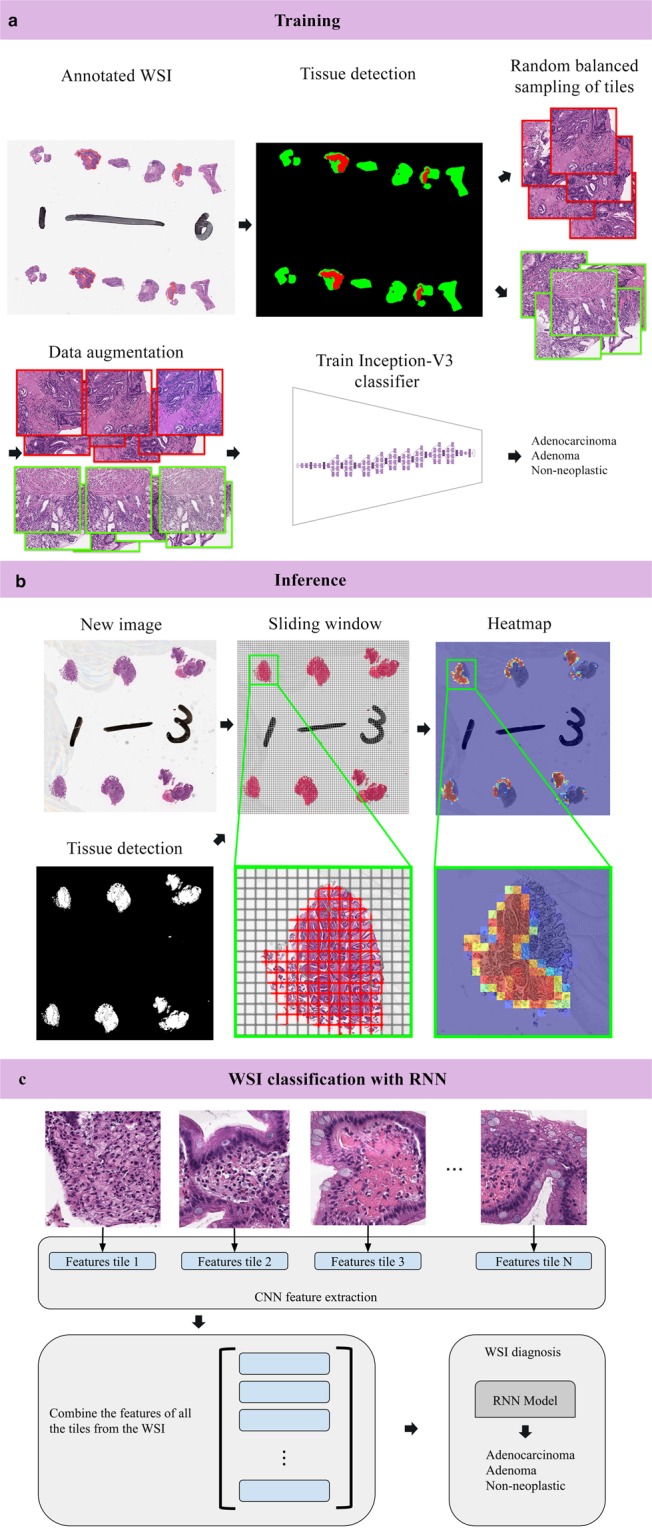
Summary of our pipeline. (**a**) Starting with annotated WSIs from the training set, tissue detection was applied, and then followed by random balanced sampling of 512 × 512 tiles from each label. Data augmentation was used during training to make the model more robust to color shifts and different orientations. All the tiles were used to train an inception v3 network to classify tiles into 3 labels: adenocarcinoma, adenoma, and non-neoplastic. (**b**) An example of inference on a new WSI. The tissue was first detected by thresholding; then, a sliding window on the detected tissue was used to extract overlapping 512 × 512 tiles which were used as input for the network. This resulted in a heatmap image. (**c**) WSI classification using an RNN involved an initial step of extracting sets of deep CNN features from each WSI in the training set to train the RNN models. A WSI classification for new WSI was then obtained by applying the trained RNN model to the WSI’s set of CNN features.

WSI classifications were obtained using two aggregation strategies: max-pooling, where a WSI is assigned the label with the maximum probability from all of its tiles, and an RNN model, which was trained to combine the information from all of the tiles using deep CNN features as input. Figure 1 gives an overview of our method. For each aggregation strategy we computed the log loss on the test sets. A high value for the log loss indicates that a model is making incorrect predictions with high confidence.

### High AUC performance of WSI evaluation of stomach and colon histopathology images

**Figure 2:**
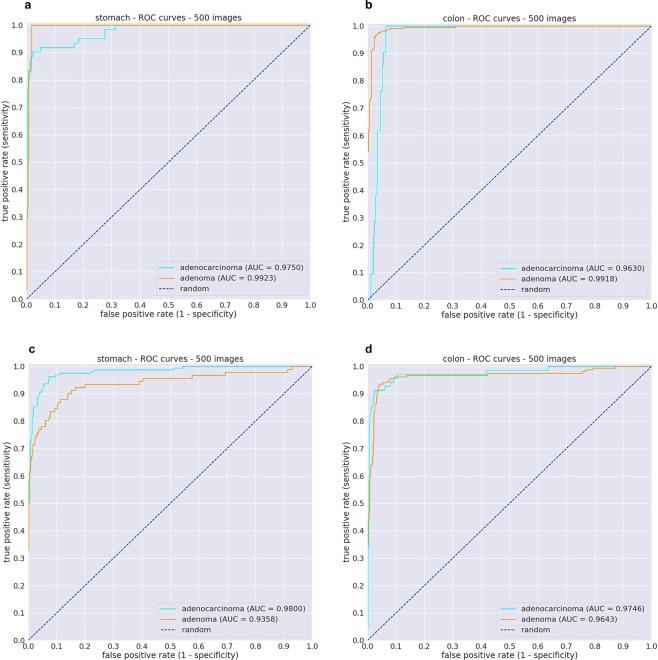
Evaluation of models on test cases obtained from different medical institution using RNN-aggr for WSI classification. (**a**,**b**) ROC curves for the stomach and colon models using 500 test cases each from the Haradoi Hospital cohort. (**c**,**d**) ROC curves for the stomach and colon using 500 test cases each from the Hiroshima University Hospital cohort.

We evaluated each of the stomach and colon models on their respective test sets from the Hiroshima University Hospital cohorts (same source as the WSI in the training set) using two aggregations methods for WSI classification: max-pooling (MP-aggr) and RNN (RNN-aggr). ROC curves and their corresponding Area Under the Curve (AUC) are summarised in Fig. 2c,d, and Table 1.

**Table 1:** ROC Area under the curve (AUC) results for the stomach and colon models when applied on the Hiroshima University Hospital cohorts using two aggregations methods for WSI classification: max-pooling (MP-aggr) and RNN (RNN-aggr).

Label	Stomach		Colon	
	RNN-aggr	MP-aggr	RNN-aggr	MP-aggr
Adenocarcinoma	0.980 (0.966–0.990)	0.966 (0.947–0.978)	0.975 (0.948–0.993)	0.972 (0.950–0.987)
Adenoma	0.936 (0.894–0.969)	0.928 (0.886–0.959)	0.964 (0.940–0.984)	0.961 (0.940–0.978)

The values between parenthesis indicate the 95% Confidence Intervals (CI)s.

A Delong test showed no statistical significance for differences in AUCs for the stomach model (adenocarcinoma (p = 0.427) and adenoma (p = 0.575)) and for the colon model (adenocarcinoma (p = 0.409) and adenoma (p = 0.945)). However, there were statistically significant differences in the values of the log losses. Table 2 summarises the log losses and their confidence intervals.

**Table 2:** Log losses for the stomach and colon models when applied on the Hiroshima University Hospital cohorts using two aggregations methods for WSI classification: max-pooling (MP-aggr) and RNN (RNN-aggr).

Label	Stomach		Colon	
	RNN-aggr	MP-aggr	RNN-aggr	MP-aggr
Adenocarcinoma	0.259 (0.175–0.368)	1.264 (1.096–1.48)	0.171 (CI, 0.113–0.243)	0.830 (0.687–1.02)
Adenoma	0.275 (0.179–0.355)	1.654 (1.348–1.82)	0.241 (CI, 0.155–0.344)	0.895 (0.714–1.08)

The values between parenthesis indicate the 95% Confidence Intervals (CI)s.

### Evaluation on an independent test set from a different medical institution

One of the primary concerns is whether the models would generalise well when applied to test sets originating from different sources. Therefore, we evaluated our models on independent test sets obtained from Haradoi Hospital, a different medical institution. ROC curves and their corresponding Area Under the Curve (AUC) are summarised in Fig. 2a,b, and Table 3. Table 4 summarises the log loss values of the models.

**Table 3:** ROC Area under the curve (AUC) results for the stomach and colon models when applied on the independent test sets obtained from Haradoi Hospital using two aggregations methods for WSI classification: max-pooling (MP-aggr) and RNN (RNN-aggr).

Label	Stomach		Colon	
	RNN-aggr	MP-aggr	RNN-aggr	MP-aggr
Adenocarcinoma	0.974 (0.952–0.994)	0.980 (0.969–0.993)	0.963 (0.945–0.977)	0.967 (0.943–0.984)
Adenoma	0.993 (0.986–0.998)	0.987 (0.978–0.995)	0.992 (0.986–0.997)	0.99 (0.979–0.996)

The values between parenthesis indicate the 95% Confidence Intervals (CI)s.

For the stomach model, a Delong test showed no statistical significance for differences in AUCs for adenocarcinoma (p = 0.495) and a statistical significance for adenoma (p = 0.5e-5). For the colon model, a statistical significance was found for adenocarcinoma (p = 0.041), and none for adenoma (p = 0.105).

### Performance of models on independent TCGA test set containing surgical sections

To further evaluate the generalisation and limitations of our models, we tested them on independent test sets obtained from TCGA. TCGA only contains surgical sections and the models were trained on biopsy specimens. As surgical resection tissues are much larger than biopsies, and given that the RNN models were trained on smaller CNN feature sets from biopsies, we only use the MP-agg method for WSI image classification.

**Table 4:** Log losses for the stomach and colon models when applied on the independent test sets obtained from Haradoi Hospital using two aggregations methods for WSI classification: max-pooling (MP-aggr) and RNN (RNN-aggr).

Label	Stomach		Colon	
	RNN-aggr	MP-aggr	RNN-aggr	MP-aggr
Adenocarcinoma	0.260 (0.173–0.338)	1.436 (1.256–1.59)	0.533 (0.390–0.688)	1.445 (1.238–1.62)
Adenoma	0.104 (0.053–0.153)	1.339 (1.085–1.65)	0.172 (0.102–0.242)	0.464 (0.327–0.557)

The values between parenthesis indicate the 95% Confidence Intervals (CI)s.

**Figure 3:**
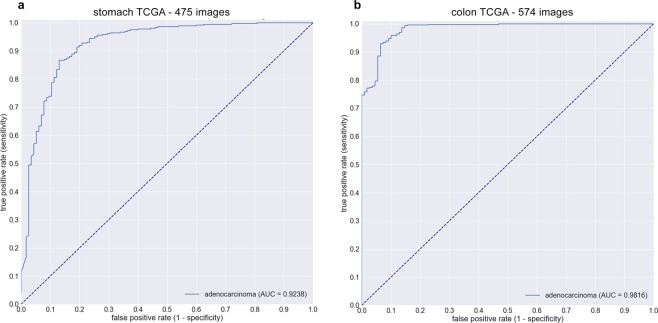
Adenocarcinoma ROC curves of stomach (**a**) and colon (**b**) models on surgical test cases obtained from TCGA using MP-aggr for WSI classification. Even though the models were trained on biopsy section, they still demonstrate generalisation on surgical sections.

For stomach, we used 475 slides from TCGA-STAD project: 360 formalin-fixed paraffin-embedded (FFPE) diagnostic samples of adenocarcinoma, and 115 flash frozen samples of normal tissue. There were no normal tissue FFPE samples available on TCGA. The AUC for adenocarcinoma classification was 0.924 (CI, 0.887–0.952) (see Fig. 3a). For colon, we used 547 slides from TCGA-COAD project: 438 FFPE adenocarcinoma samples, and 109 flash frozen normal tissue samples. The AUC for adenocarcinoma classification was 0.982 (CI, 0.968–0.991) (see Fig. 3b).

### Comparison of stomach model against a group of pathologists under time constraints

**Figure 4:**
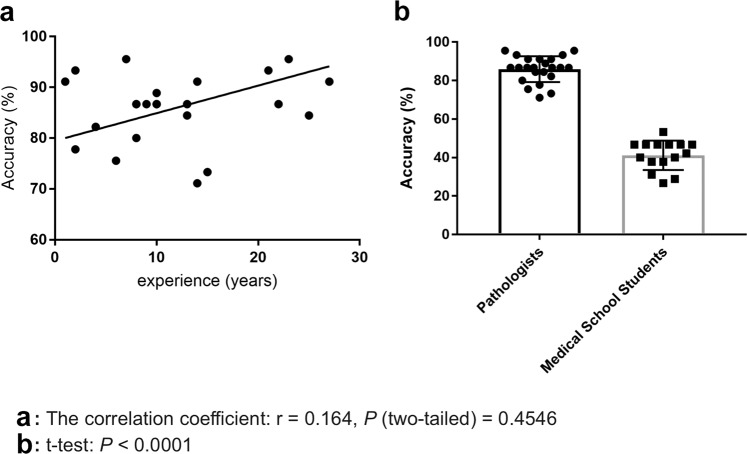
The diagnostic accuracy (%) of pathologists and medical school students under time constraints for 45 test cases (15 adenocarcinoma, 15 adenoma, and 15 non-neoplastic). (**a**) The accuracy as function the the pathologists’ experience in years. No statistical significance (p = 0.4546) was found between pathologists based on years of experience. (**b**) Boxplots comparing the diagnostic accuracy of pathologists and medical school students. High statistical significance was found between pathologists and medical students (p < 0.0001).

**Figure 5:**
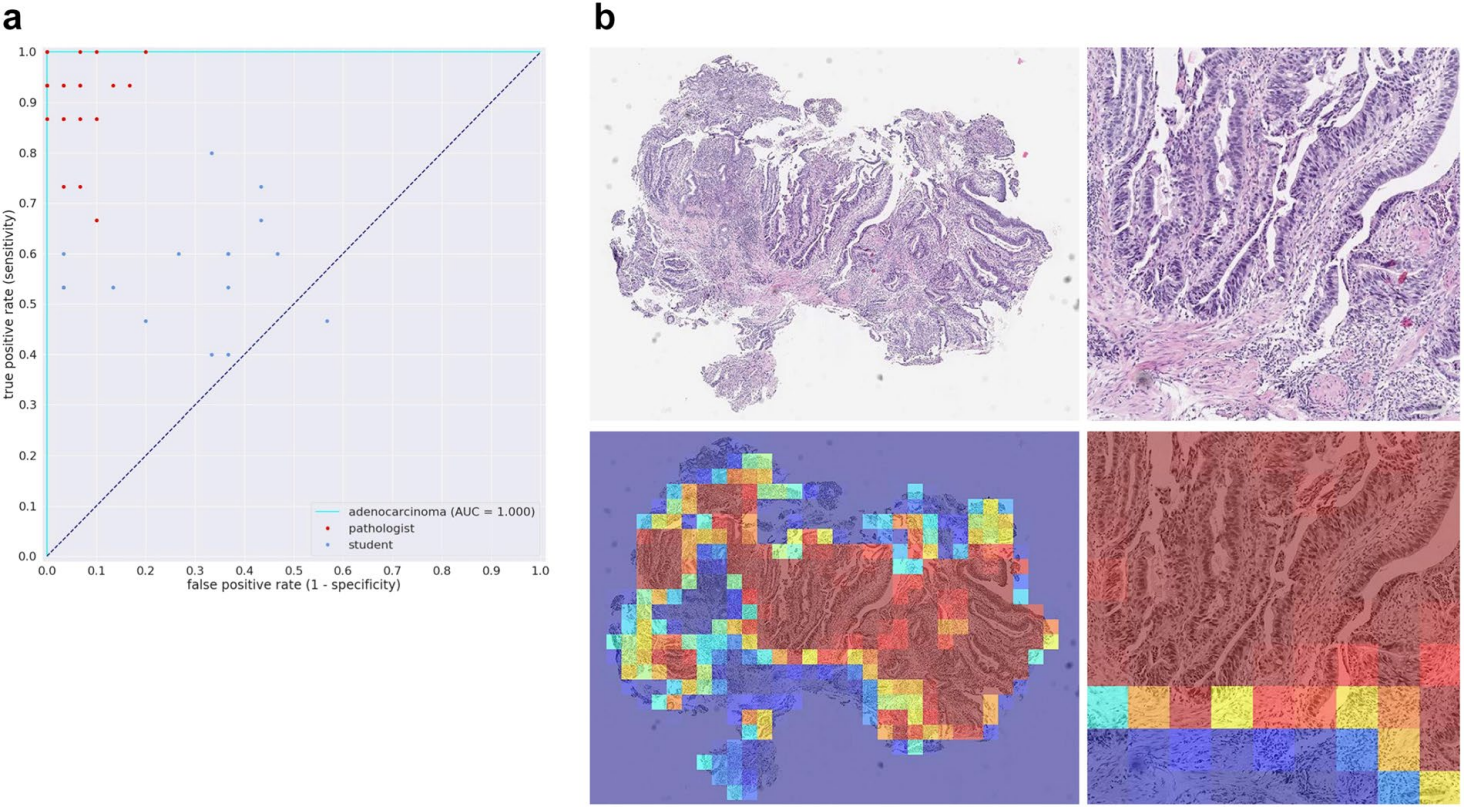
(**a**) Stomach adenocarcinoma ROC curve for the 45 test cases used for comparison against a group of pathologists and medical students. (**b**) Example heatmap output from the stomach model for adenocarcinoma using a stride of 128 × 128.

**Figure 6:**
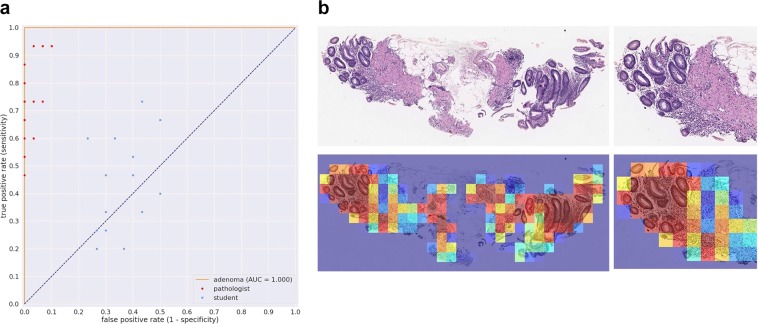
(**a**) Stomach adenoma ROC curve for the 45 test cases used for comparison against a group of pathologists and medical students. (**b**) Example heatmap output from the stomach model for adenoma using a stride of 128 × 128.

**Figure 7:**
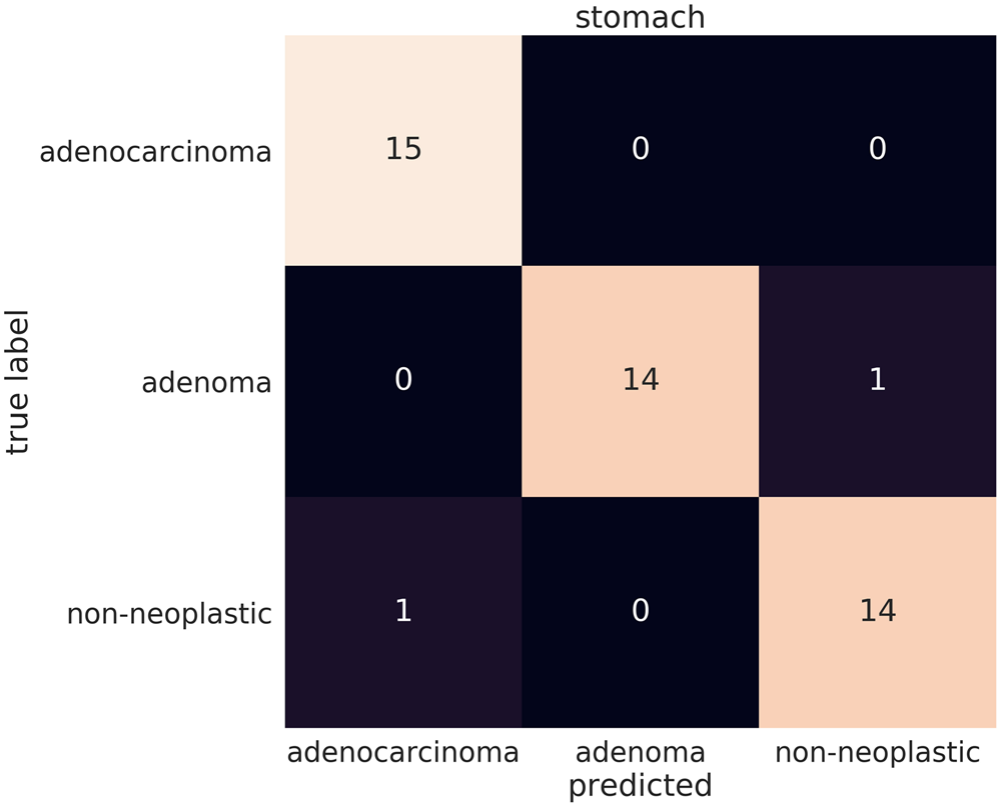
Confusion matrix of the stomach model for WSI image classification into adenocarcinoma, adenoma, and non-neoplastic on the 45 test cases that were used assess the performance of the pathologists under time constraints.

We evaluated our stomach model against a group of pathologists and medical students volunteers that were not involved in annotating the training set using a test set of 45 images, consisting of 15 WSI of adenocarcinoma, 15 of adenoma, and 15 of non-neoplastic lesions. The ultimate aim of using deep learning models in a clinical workflow is the speed at which they can operate; the classification time for WSI using the trained model ranged from 5 to 30 seconds, depending on the size of the WSI. We therefore performed the comparison under time constraints. Each pathologist was presented with the 45 images in a random order and had only 30 seconds to make a decision on the diagnosis. All pathologists observed 45 WSIs without using conventional microscopes. The average accuracy (percentage of correct diagnoses) achieved by pathologists was 85.89% ± 1.401%, (n = 23), while that of medical school students was 41.19% ± 1.974%, (n = 15). A random diagnosis would have an accuracy of 33.33%. The stomach model achieved an accuracy of 95.6% when using the standard probability threshold of 0.5. Figure 4 compares the performances of the pathologists and medical students. No statistical significance (p = 0.4546) was found between pathologists based on years of experience. Figures 5a and 6a compare the performance of the models against 23 pathologists and 15 medical school students. Figures 5b and 6b show example heatmap outputs. Figure 7 shows the confusion matrix of the stomach model.

## Discussion

In this work, we trained deep learning models to classify epithelial tumours in biopsy WSIs of stomach and colon. Each model was trained on WSI obtained from a single medical institution and was evaluated on two independent test sets originating from the same and from a different institution, respectively. On a given WSI, the output of the models were a set of heatmaps that allowed visualising the locations of the predicted labels and a primary classification for the WSI. We think that the results of this study are important as, to the best of our knowledge, there have been no previously reported studies that were validated on large datasets, especially for gastric and colonic epithelial lesions.

There were mostly no statistical differences between the two aggregation methods on the different test sets, and, when there was, the RNN-aggr was statistically better. However, there were statistically significant differences in log losses, with the RNN-aggr having a much lower log loss compared to the MP-aggr. This meant that the WSI probabilities using MP-aggr were much higher requiring a high cut-off thresholds (around 0.99) for obtaining a classification, while the cut-off thresholds with RNN-aggr were closer to the standard 0.5 probability. This is somewhat to be expected as the MP-aggr is more prone to errors as a single WSI can contain up to thousands of tiles, and all it would take is one false positive tile to result in an incorrect classification. This was also noted by Campanella *et al*.^17^. This is especially a problem given that the annotations were not done on a detailed cell level with some of the tumour annotated regions containing areas of inflammatory cell infiltration. On the other hand, RNN-aggr considers all the tiles in the slide before outputting a classification and can suppress false positives.

All of the slides for training were exclusively obtained from Hiroshima University Hospital. This is a potential limitation as the single institution cohort may not represent the full diversity and heterogeneity of samples that may be encountered in a clinical setting from a variety of institutions. The performance on the independent test sets from Haradoi Hospital showed no degradation in performance which is highly promising. In addition, the evaluation on the independent test sets from TCGA using a mix of FFPE and flash frozen tissue samples demonstrated very promising results for the generalisation of predicting adenocarcinoma on surgical resection cases even though the models were trained mostly on biopsies. The colon model performed better than the stomach model on TCGA, which is most likely due to the colon training set containing some surgical resection cases. We were unable to validate the adenoma prediction as there were none available from TCGA.

The conditions imposed on the pathologists in this study to perform under time constraints are unrealistic in clinical practice, as given enough time and exhaustive resources to pathologists, they would achieve 100% accuracy. However, this does show the primary advantage of integrating such a system in clinical workflow tool: the speed at which it can operate. It could be used as part of a integrated workflow where, as soon as the slides are scanned, a diagnosis is predicted automatically and used to rank cases based on order of priority for review by a pathologist. This would allow a pathologist to inspect first the cases that potentially require the most attention, allowing a faster turn around. The tool could be applied on a large database of WSI to organise and retrieve slides based on a given predicted diagnosis, and it could also serve as a second reader to confirm the diagnosis or alert the pathologist in case of disagreement on the primary diagnosis, requesting further inspection. Integrating AI within the computational pathology workflow would be of high benefit for easing the ever increasing workloads on pathologists, especially in regions that have shortages in access to pathological diagnosis services.

In the next step, we would to like to further classify carcinoma sub-types (e.g., papillary adenocarcinoma, tubular adenocarcinoma, mucinous adenocarcinoma, signet-ring cell carcinoma, adenosquamous carcinoma, etc), which play an important role in developing further practical aid for surgical pathologists, and to train models to predict patient outcome^26,27^. We would like to witness a future where pathologists and AI collaborate together to support the foundation of medical care.

## Methods

### Clinical cases

For the present retrospective study, 4128 cases of human gastric epithelial lesions and 4036 of colonic epithelial lesions HE (hematoxylin & eosin) stained histopathological specimens were collected from the surgical pathology files of Hiroshima University Hospital after histopathological review of those specimens by surgical pathologists. The experimental protocol was approved by the ethical board of the Hiroshima University (No. E-1316). 500 cases of human stomach epithelial lesions and 500 of colonic epithelial lesions HE stained surgical specimens were collected from and Haradoi Hospital (Fukuoka) after histopathological review and approval by surgical pathologists. All research activities were performed in accordance with relevant guidelines/regulations in the Hiroshima University Hospital and Haradoi Hospital. Informed consent to use tissue samples and pathological diagnostic reports for research purposes had previously been obtained from all patients prior to the surgical procedures at both hospitals. The test cases were selected randomly, so the obtained ratios reflected a real clinical scenario as much as possible.

### Datasets and annotations

The stomach is anatomically divided into five regions: cardia, fundus, corpus, antrum, and pylorus. These anatomic regions exhibit three types of gastric mucosa with transitional forms in the boundary areas: cardiac, fundic, and pyloric (antral). These three types of gastric gland are composed of two components: foveola and secretory portion (adenomere). Foveola is an important part of the pathogenesis of gastric malignant epithelial tumor (e.g., adenocarcinoma) because the layer of stem cells located at the base. Epithelial benign tumors (e.g., adenoma) and non-neoplastic lesions (e.g., gastritis and polyp) also occur from gastric mucosa. The colon (large bowel) is divided into six regions: cecum, ascending colon, transverse colon, descending colon, sigmoid colon, and rectum. The colonic mucosa is comprised of three components: epithelium, lamina propria, and muscularis mucosae. Colonic epithelial tumors (malignant: adenocarcinoma; benign: adenoma) and non-neoplastic lesions (e.g., colitis and polyp) occur from colonic mucosa and their epithelial cell components are originated from colonic epithelium. For the datasets obtained from Hiroshima University Hospital, the stomach dataset consisted of 4,128 WSIs, and the colon dataset consisted of 4,036 WSIs. Table 5 breaks down the distribution of the dataset into train and test sets. The stomach dataset was solely composed of biopsy images, while the colon dataset contained some surgical specimens (5%). All cases used for annotation were looked over carefully and verified by pathologist(s) prior to annotation.

**Table 5:** Distribution of WSI in the dataset.

	Stomach				Colon			
	Hiroshima cohort			Haradoi cohort	Hiroshima cohort			Haradoi cohort
	All	Train	Test	Test	All	Train	Test	Test
Adenocarcinoma	1386	1228	158	61	481	412	69	21
Adenoma	563	472	91	30	370	215	155	210
Non-neoplastic	2179	1928	251	409	3185	2909	276	269
Total	4128	3628	500	500	4036	3536	500	500

The WSIs were manually annotated by a group of surgical pathologists (annotation pathologists) who perform routine histopathological diagnoses by drawing around the areas that corresponded to one of the three labels: adenomcarcinoma, adenoma, and non-neoplastic. Adenocarcinoma annotation was performed to include both tumour parenchyma and stromata (the part of a tissue with inflammatory cell infiltration or fibrosis within the tumour). Any regions that did not contain adenocarcinoma or adenoma were included under the non-neoplastic label, which consisted of inflammatory (e.g., gastritis, colitis) and hyperplastic (e.g., fundic gland polyp, colorectal polyp) lesions as well as normal. Non-neoplastic annotation was performed to include epithelial cells and stromata. The average annotation time per WSI was about 10 minutes. Annotations performed by annotation pathologists were modified (if necessary), confirmed, and verified by another group of pathologists and used as training datasets. Each annotated WSI was seen by at least two pathologists, with the final checking and verification performed by a senior pathologist. Cases that had discrepancies in the annotation labels were excluded from training. Some WSI contained multiple annotation labels. Therefore, a single WSI label of major diagnosis was assigned to a given WSI based on the following order of priority: adenocarcinoma, adenoma, non-neoplastic. For example, if the WSI contained annotations for both adenocarcinoma and adenoma, then the WSI diagnosis was adenocarcinoma.

### Image pre-processing

We operated on a zoom level of 20X magnification. For each WSI, we first performed tissue detection to remove the large areas of white background. The tissue detection was based on a simple saturation thresholding. Based on the the obtained tissue masks, we extracted overlapping 512 × 512 tiles with different random orientations, as the tissue samples do not have a specific orientation. We sampled the same number of tiles (n = 400) from each label from a given WSI to avoid label imbalance (when there are more images of a given label compared to another label) on the level of WSI. For example, if a WSI had both adenocarcinoma and non-neoplastic, then we sampled 400 tiles of adenocarcinoma and 400 tiles of non-neoplastic. During sampling, titles were rejected if more than 50% of the annotation label fell outside a 128 × 128 centred window within the tile; otherwise, the tiles were assigned the label that they overlapped with. A total of around four million tiles were extracted from each dataset.

### Deep learning model

We used the standard inception-v3 network architecture^23^ with an input size of 512 × 512. We reduced the number of parameters by using a depth multiplier of 0.35 resulting in a slimmed-down version of the architecture with a reduced number of weight parameters; the depth multiplier is a scalar factor that is used to scale the depth of all the convolutional layers simultaneously. This allowed speeding up training and inference with minimal impact on prediction accuracy. Slimmed-down versions of the inception architecture using a depth multiplier as low as 0.1 have been observed to achieve similar performance to the full version of the network by Liu *et al*.^25^ on breast cancer WSI. We did not do any further tuning to the network. We trained the slimmed-down network from scratch.

To make the algorithm more robust against image variations, and add a regularisation effect, we applied several data augmentation techniques. This included techniques such as randomly flipping the images left-right or up-down with additional random rotations; minor scaling; minor position offsets; hue and saturation offsets; and contrast and brightness perturbations. The images were then scaled to a floating value range of [−1.0, 1.0].

While we sampled the tiles from each label equally from a given WSI, the dataset still contained an unequal number of WSIs for each label, resulting in an overall unequal number of extracted tiles for each label. To account for this imbalance, we used a weighted cross entropy loss function. The weights were used to reduce the label imbalance on a WSI level so that the network does not favour predicting one label over the other. We trained the network using the Adam optimisation algorithm^28^ with a momentum of 0.9, decay of 0.9, epsilon of 1.0. We used a decaying learning rate with a warm start, with a minimum starting learning rate of 0.001 and a maximum learning rate of 0.05. We used a batch size of 128, and the training was run for a total of 625 K iterations. The model with the lowest validation error was chosen as the final model.

### RNN model for whole-slide classification

The previously trained inception-v3 network can be used as a feature extractor by removing the final fully-connected classification layer. The output of the inception-v3 feature extractor, with depth multiplier 0.35, is a 715 feature vector. Tiles were extracted from a WSI using a sliding window with a stride of 256 × 256 pixels. During inference, all the tiles from a given WSI need to be checked in order to make a WSI classification.

An RNN model can take an arbitrary length sequence and produce a single output^29^. Each slide had an arbitrary number of tiles extracted from the tissue regions, and we used all of the tiles as input to the RNN model. During training, the order of the features of the tiles were randomly shuffled at each step to reduce dependency on tile input order. We used an RNN consisting of two Long Short-Term Memory (LSTM)^30^ layers with a hidden state representation size of 128. We trained the model using stochastic gradient descent with a batch size of one. The model was trained with a learning rate of 0.001 with a decay of 1e-6 for 50 epochs. The best performing model on the 5% validation subset was picked as the final model.

### Software and statistical analysis

The models were implemented and trained using TensorFlow^31^. AUCs were calculated in python using the scikit-learn package^32^ and plotted using matplotlib^33^. The 95% CIs of the AUCs were estimated using the bootstrap method^34^ with 1000 iterations. Pairs of AUCs were compared using the two-tailed DeLong’s test^35^ for two correlated ROC curves. Pairs of log losses were compared using the paired two-sided student t-test. Correlation analysis was performed between the pathologists’ experience in years and accuracy. A Student t-test was used to examine differences in the diagnostic accuracy of pathologists and medical school students. GraphPad Prism version 7.04 (www.graphpad.com) was used to generate Fig. 4.

## Data Availability

The majority of datasets used in this study are not publicly available due to specific institutional requirements governing privacy protection. Supporting data for this study were made available to Editorial Board Members and referees at the time of submission for the purposes of evaluating the manuscript. The external TCGA datasets were obtained from the TCGA-STAD and TCGA-COAD projects and are publicly available through the Genomic Data Commons portal (https://portal.gdc.cancer.gov/).
